# Histatin 5 inhibits adhesion of *C. albicans* to Reconstructed Human Oral Epithelium

**DOI:** 10.3389/fmicb.2015.00885

**Published:** 2015-08-28

**Authors:** Eduardo B. Moffa, Maria C. M. Mussi, Yizhi Xiao, Saulo S. Garrido, Maria A. A. M. Machado, Eunice T. Giampaolo, Walter L. Siqueira

**Affiliations:** ^1^The University of Western Ontario – Department of Biochemistry and Schulich Dentistry, Schulich School of Medicine and Dentistry, London, ONCanada; ^2^Department of Dental Materials and Prosthodontics, Araraquara Dental School – Universidade Estadual Paulista, São PauloBrazil; ^3^School of Dentistry, University of São Paulo, São PauloBrazil; ^4^Department of Biochemistry and Technological Chemistry – Institute of Chemistry – Universidade Estadual Paulista, São PauloBrazil; ^5^Department of Pediatric Dentistry, Orthodontics and Public Health, Bauru Dental School, University of São Paulo, BauruBrazil

**Keywords:** histatins, salivary proteins, mucosal pellicle, oral mucosa, *Candida albicans*

## Abstract

*Candida albicans* is the most pathogenic fungal species, commonly colonizing on human mucosal surfaces. As a polymorphic species, *C. albicans* is capable of switching between yeast and hyphal forms, causing an array of mucosal and disseminated infections with high mortality. While the yeast form is most commonly associated with systemic disease, the hyphae are more adept at adhering to and penetrating host tissue and are therefore frequently observed in mucosal fungal infections, most commonly oral candidiasis. The formation of a saliva-derived protein pellicle on the mucosa surface can provide protection against *C. albicans* on oral epithelial cells, and narrow information is available on the mucosal pellicle composition. Histatins are one of the most abundant salivary proteins and presents antifungal and antibacterial activities against many species of the oral microbiota, however, its presence has never been studied in oral mucosa pellicle. The objective of this study was to evaluate the potential of histatin 5 to protect the Human Oral Epithelium against *C. albicans* adhesion. Human Oral Epithelial Tissues (HOET) were incubated with PBS containing histatin 5 for 2 h, followed by incubation with *C. albicans* for 1 h at 37°C. The tissues were then washed several times in PBS, transferred to fresh RPMI and incubated for 16 h at 37°C at 5% CO_2_. HOET were then prepared for histopathological analysis using light microscopy. In addition, the TUNEL assay was employed to evaluate the apoptosis of epithelial cells using fluorescent microscopy. HOET pre-incubated with histatin 5 showed a lower rate of *C. albicans* growth and cell apoptosis when compared to the control groups (HOET alone and HOET incubated with *C. albicans*). The data suggest that the coating with histatin 5 is able to reduce *C. albicans* colonization on epithelial cell surfaces and also protect the basal cell layers from undergoing apoptosis.

## Introduction

Removable dentures provide edentulous patients with the rehabilitation of masticatory and esthetic functions (Dhir et al., 2007); however, one consequence of the continual use of dentures is the adhesion of microorganisms and biofilm formation (Lazarin et al., 2014). *Candida* sp. are opportunistic pathogens that are frequently isolated from the oral cavity, and its biofilms are often associated with candidiasis (Gomes et al., 2011; Hahnel et al., 2012; Lazarin et al., 2014). These biofilms are extremely resistant to antimicrobial agents compared to planktonic microorganisms due to the presence of extracellular polymeric substance (EPS) generated by microorganisms themselves, which acts as an impervious and protective covering of biofilms. They are not only resistant to the action of most of the available antifungal substances, but they also resist the phagocytic action of our immune cells (Chandra et al., 2001b). Salivary pellicle is a thin, biological film of selective salivary proteins, lipids, and carbohydrates which coats oral surfaces and acts as an interface between the oral surface and the first layer of microorganisms. When the salivary proteins adsorb on the tooth surface, it is called acquired enamel pellicle (AEP). A mature AEP has more than 130 different proteins, ranging from protein to peptide size (Siqueira et al., 2007). The formation of a saliva-derived protein pellicle on the mucosa surface can provide protection against the colonization and invasion of *Candida albicans* on oral epithelial cells, which leads to candidiasis. In the literature, narrow information is available on the mucosal pellicle composition, and few studies have reported the presence of salivary proteins such as mucins, cystatins, IgA, amylase, and statherin (Bradway et al., 1992; Gibbins et al., 2013) on the oral epithelial cell.

Histatins are one of the most abundant salivary proteins and have been shown to be multifunctional in the oral cavity due to their strong antifungal and antibacterial activities against many species of the oral microbiota (Pollock et al., 1984). Histatin 5, for example, adsorbed as a protein integument on PMMA and hydroxyapatite, effectively inhibits *C. albicans* colonization (Vukosavljevic et al., 2012). Potentially, histatin 5 could be one of the salivary components in mucosal pellicle that protects the oral cavity against infections caused by pathogenic microorganisms. The purpose of this study was to assess the potential effect of histatin 5 human oral mucosa coating to protect epithelial cells against *C. albicans* colonization.

## Materials and Methods

### Microorganisms and Growing Conditions

Stock culture of *C. albicans* (ATCC 90028) maintained at –80°C was used in each experiment. After recovery, *C. albicans* was maintained on Sabouraud Dextrose Agar (SDA; BDTM Difco, Franklin Lakes, NJ, USA), stored at 4°C. A loopful of the stock culture of *C. albicans* was streaked onto SDA and incubated aerobically at 37°C for 48 h to prepare the yeast inoculum. One loopful of this young culture was then transferred to 20 mL of YNB broth (BioShop^®^, Canada Inc., Burlington, ON, Canada) supplemented with 50 mM glucose and incubated at 37°C for 21 h. Cells of the resultant culture were harvested, washed twice with PBS at pH 7.4, and centrifuged at 4,000 × *g* for 10 min. The final concentration was adjusted to 10^7^ cells mL^-1^ (Chandra et al., 2001a; Pereira-Cenci et al., 2008).

### *Candida albicans* Killing Assay

A total of 50 μL from the suspension was added to 50 μL of a 10-fold serial dilution of histatin 5 in a 96-well polystyrene microtiter plate (Corning Inc., Corning, NY, USA) with an initial concentration of 800 μg/mL. For the control group, 50 μL of *C. albicans* suspension was added to 50 μL of PBS. After 1.5 h of incubation at 37°C, 50 μL of suspension from the selected wells were diluted in 9 mL of PBS. After that, 25 μL aliquot of the diluted suspension was plated on SDA and incubated at 30°C for 48 h (Helmerhorst et al., 2006). Colony counting was used to assess cell viability (CFU mL^-1^). This experiment was carried out in triplicate.

### The Effect of Histatin 5 When Adsorpted on a Microtiter Plate Prior C. *albicans* Biofilm Formation

Prior to the *C. albicans* assay, histatin 5 (protein purity > 95%, GenScript, Piscataway, NJ, USA) was re-suspended in distilled water with a concentration of 15 μg/mL. A total volume of 200 μL of histatin 5 solution was added to each well of a 96-well polystyrene microtiter plate, and the wells were incubated for 2 h at 37°C under gentle agitation. The wells were then washed with distilled water to remove the non-adsorbed histatin 5, and subsequently used for the formation of *C. albicans* biofilm at different time periods: 90 min, 24 h, 48 h, and 72 h. Non-adherent *C. albicans* cells were removed by washing them with PBS. At each time period, the adherent cells were harvested from the microtiter plate and plated onto SDA as described above. This experiment was carried out in triplicate.

### Effect of Histatin 5 When Incubated Over a 48 h *C. albicans* Biofilm Formation

A 48 h *C. albicans* biofilm was developed as described above. The only difference was the absence of histatin 5 as a solid surface. After the *C. albicans* biofilms formation, histatin 5 was added with different concentrations, ranging from 6.3 to 12,800 μg/mL. After 24 h of contact with histatin 5, the *C. albicans* were washed three times with PBS and the cells were harvested, which was followed by 10-fold serial dilutions from 10^-1^ to 10^-4^ and plated onto SDA. The experiment was carried out in triplicate.

For the three tests described above, the number of CFU mL^-1^ was calculated and the analyses of variance (ANOVA) follow by the Tukey Honestly Significant Difference (HSD) *post hoc* test was used to determine differences between means (*a* = 0.05).

### Cell Culture

The cytotoxicity effect of histatin 5 was evaluated on gingival fibroblasts grown in Dulbeccco’s Modified Eagle Medium (gibco^®^ by life technologies), supplemented with antibiotic–antimycotic solution (Sigma–Aldrich) and 10% v/v fetal bovine serum (gibco^®^ by life technologies). The culture was maintained at 37°C in an atmosphere of 5% CO_2_ in 95% air. (Thermo Scientific, USA). Cells were cultured until reaching confluence (90%) and removed with trypsin (0.05%)/EDTA (0.02%), (gibco^®^ by life technologies) in 1X PBS. The trypsin was inactivated by the addition of culture medium, and the cells were then subjected to centrifugation at 2000 rpm for 5 min, resuspended and re-plated. The medium was changed two to three times per week. Total viable cell counts were made in a Neubauer chamber (New Optics), and a suspension containing 2.0 × 10^4^ cells/ml was placed in 24 well plates (TPP) and incubated in a humidified atmosphere containing 5% CO_2_ at 37°C for 48 h. After the incubation period, the culture medium was disposed, and attached cells remained at the bottom of the plates. A serial dilution of histatin 5 was performed using fresh culture medium, and 1 mL was added to each well. The plates were maintained at 37°C in an atmosphere of 5% CO_2_ in 95% air for 24 h. Five wells were used for each experimental group. Five wells received only 1 ml of culture medium Dulbeccco’s Modified Eagle Medium (gibco^®^ by life technologies), supplemented with antibiotic–antimycotic solution (Sigma–Aldrich) and 10% v/v fetal bovine serum (gibco^®^ by life technologies), which served as the negative control.

### Cytotoxicity Assay (MTT)

Mitochondrial dehydrogenase activity was measured using the MTT [3-(4,5-dimethylthiazol-2-yl)-2,5-diphenyltetrazolium bromide] assay from Sigma–Aldrich. After 24 h of cell growth in either control or test culture media, 100 μl of MTT stock solution (MTT, Sigma Chemical Co., St. Louis, MO, USA) was added to each well. The plates were incubated for 4 h at 37°C in 5% CO_2_. After the incubation period, the cultures were removed from the incubator, and the resulting formazan crystals were dissolved by adding 100 μL of MTT solubilization solution (MTT, Sigma Chemical Co., St. Louis, MO, USA). Plates were then shaken until the crystals were completely dissolved and the absorbance was spectrophotometrically measured at a wavelength of 570 nm (Labsystems Multiscan Ascent, Thermo Labsystems, Vantaa, Finland). All experiments were performed three times.

The results were submitted to ANOVA and Bonferroni tests. In addition, the results were also evaluated in accordance with ISO standard 10993-5:

0: not cytotoxic (inhibition below 25%)
1: slightly cytotoxic (inhibition between 25 and 50%)
2: moderately cytotoxic (inhibition between 50 and 75%)
3: intensely cytotoxic (inhibition higher than 75%).

### Human Oral Epithelium Infection

Reconstructed human oral epithelium (HOE) tissues (SkinEthic Laboratories, Lyon, France) were incubated overnight in 12-well polystyrene microtiter plates containing 1.0 mL of Maintenance Medium at 37°C in a humidified atmosphere with 5% CO_2_. After the incubation period, the tissues were washed three times with PBS. Before the infection procedure, the HOE tissues were divided into four experimental groups (*n* = 3), with G1 and G2 incubated with PBS, and G3 and G4 incubated with histatin 5 (50 μg mL^-1^). The incubation time was performed for 2 h at 37°C under 5% CO_2_ (Vukosavljevic et al., 2012). Following, the tissues were washed three times with PBS and then transferred to a new microtiter plate containing 5 mL of RPMI-1640 medium (gibco^®^ by life technologies) in each well. For G2 and G4, aliquots of 50 μL of 10^7^ mL^-1^ C. *albicans* cells were transferred into each well and incubated for 60 min at 37°C in an orbital shaker at 75 rev min^-1^ for the adhesion phase. Tissues were then washed three times with PBS, transferred to a new microtiter plate filled with fresh RPMI-1640 medium and incubated for 24 h at 37°C with 5% CO_2_. The same protocol was followed for G1 and G3 but without adding the microorganism.

### Histology and Light Microscopy

The HOE tissues were excised around the circumference with a blade, fixed immediately in 4% buffered formalin, and then embedded in paraffin wax. For each HOE tissue, 30 of 3 μm paraffin wax sections were prepared. After deparaffinized in xylene, the sections were stained using hematoxylin and eosin (H&E) technique, mounted in DPX mountant (VWR, Lutterworth, UK), and examined by light microscopy with a Leica DFC295 camera connected to a Leica DM1000 microscope (Leica Microsystems, Wetzlar, Germany). To determine which cells were in apoptosis, terminal deoxynucleotidyl transferase dUTP nick end labeling (TUNEL) was assayed fluorescently using *in situ* cell death detection kit (Roche, West Sussex, UK) and HOE sections were imaged under the fluorescent microscope Zeiss Axio Imager M.1 Axio coupled with a Qimaging Retiga EXi CCD camera (Zeiss, Jena, Germany). Histological changes during infection were examined by microscopy at ×40 magnification.

## Results

**FIGURE 1:**
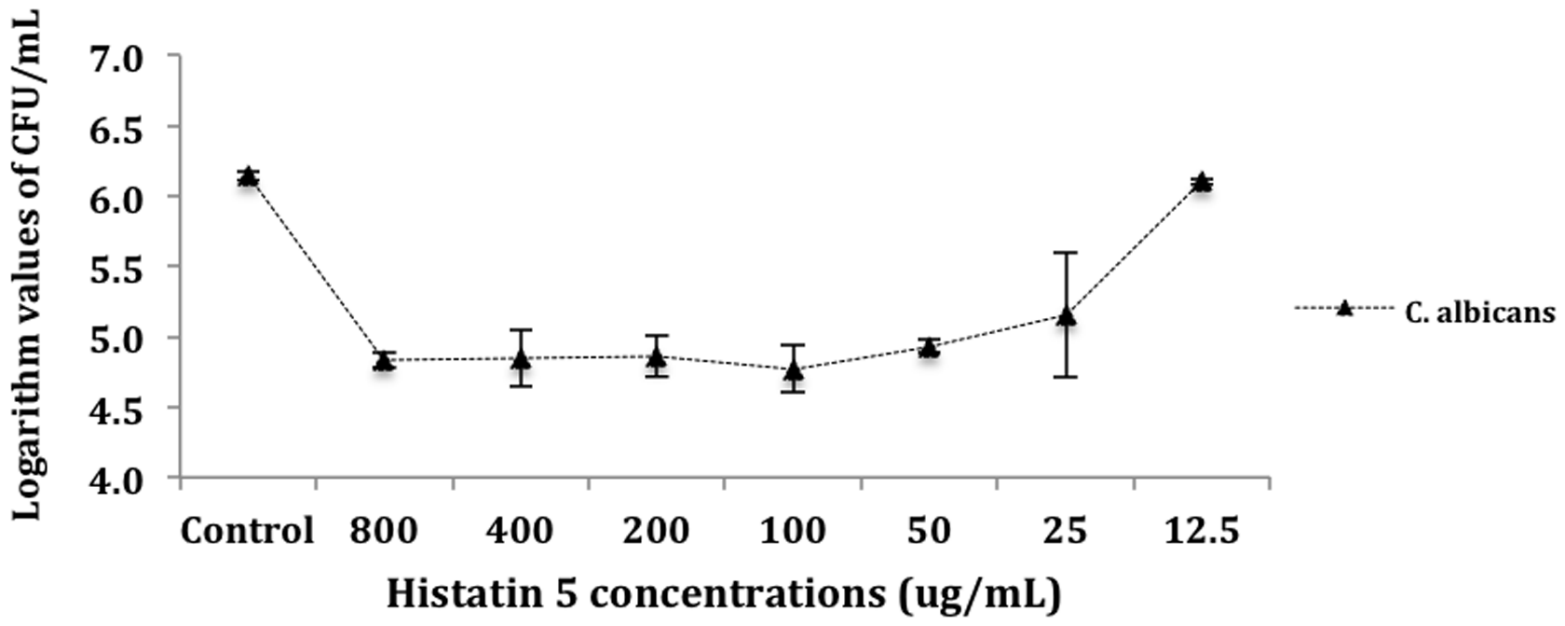
**Killing of *Candida albicans* by histatin 5.** Cells were incubated for 1.5 h at 37°C with a dilution series of histatin 5 or without histatin 5 (control group). The dilutions were then plated in Sabouraud Dextrose Agar (SDA) media and the logarithm values of CFU/mL^-1^ were calculated.

For the purposes of analysis, CFU mL^-1^ values were transformed into logarithm values (log10). The results of number of colony-forming units per milliliter (CFU mL^-1^) were evaluated statistically by ANOVA. Tukey HSD *post hoc* test was used to determine differences between means (*a* = 0.05). **Figure 1** presents the mean (M) and standard deviation (SD) of the logarithm cell count in CFU/mL for the killing assay test. There were no statistically significant differences between the control and the group treated with 12.5 μg/mL of histatin 5. Moreover, the control and the group treated with 12.5 μg/mL of histatin 5 produced higher mean values of logarithm cell count. This was statistically different (*p* = 0.00) when compared to the other groups, which are statistically similar to each other (**Figure 1**).

**FIGURE 2:**
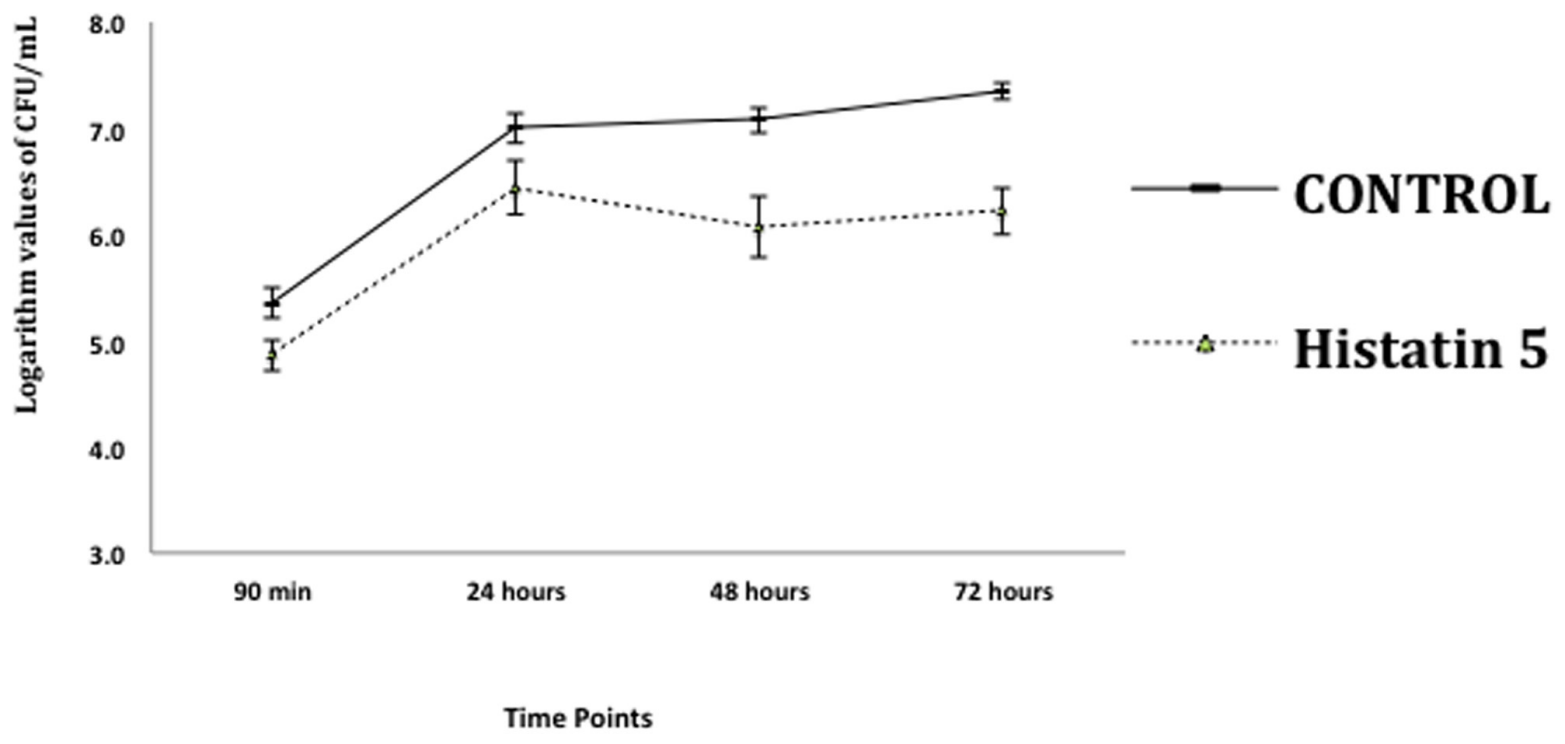
**Logarithm values of CFU/mL^-1^ when histatin 5, in a concentration of 15 μg/mL, is adsorpted on a microtiter plate prior to *C. albicans* biofilm formation in different stages of growth: 90 min, 24, 48 and 72 h, and their respective controls**.

**FIGURE 3:**
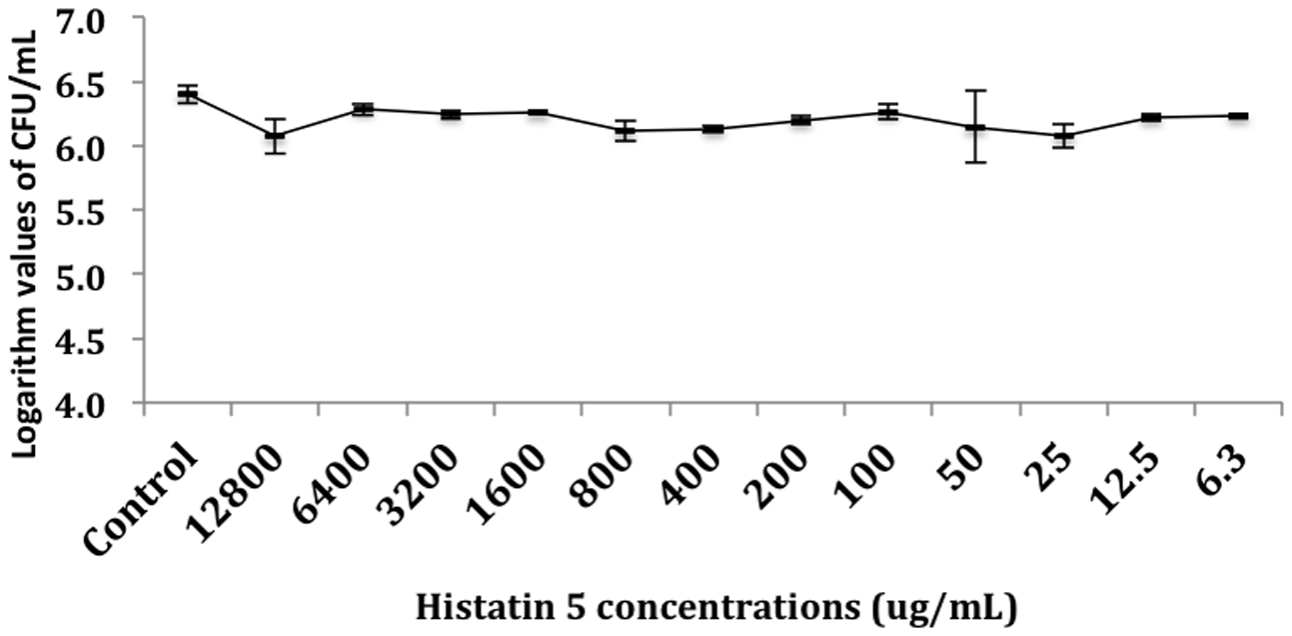
**Logarithm values of CFU/mL^-1^ when histatin 5 was in different concentrations, ranged from 12,800 to 6.3 μg/mL, were incubated for 24 h over a 48 h *C. albicans* biofilm**.

The effect of histatin 5 when adsorbed to microtiter plate prior *C. albicans* biofilm formation was summarized in **Figure 2**. The logarithm cell counts in CFU/mL were compared with the two groups (control and group coated with histatin 5) at each time point. The results revealed that the group coated with histatin 5 in a concentration of 15 μg/mL previous to microorganisms colonization exhibited lower mean values of *C. albicans* for all time points (90 min, 24 h, 48 h, and 72 h) when compared with control group (*p* = 0.00). On the other hand, according to the results presented in **Figure 3**, when histatin 5 was incubated for 24 h over a prior formed 48 h *C. albicans* biofilm, there was no difference in cell counts in all tested concentrations of histatin 5 (6.3–12,800 μg/mL) when compared with each other and with the control (*p* = 0.454).

The ANOVA followed by Bonferroni test shows statistically significant differences between groups, where we can observe that the concentrations of 12800, 6400, and 3200 ug/mL are statistically similar and have the highest cytotoxicity by reducing the viability of the fibroblasts to up to 94%. The results show that concentrations ranging from 800 to 6.25 μg/mL have statistically similar values to the control group, may be considered non-cytotoxicity to human cells (**Table 1**).

**Table 1:** Mitochondrial dehydrogenase activity of gingival fibroblast exposed to histatin 5 solutions.

Group	Mean	SD	% of inhibition
Control	0.230^AB^	0.043	0
12800	0.015^C^	0.002	94
6400	0.015^C^	0.001	94
3200	0.025^C^	0.003	89
1600	0.173^B^	0.008	25
800	0.199^AB^	0.013	14
400	0.220^AB^	0.021	4
200	0.211^AB^	0.025	8
100	0.231^A^	0.017	0
50	0.204^AB^	0.026	9
25	0.142^AB^	0.020	7
12.5	0.221^AB^	0.075	4
6.25	0.208^AB^	0.076	9

*Cytotoxicity mean values designated with vertically identical capital letters were not statistically different (*P* > 0.05).*

**FIGURE 4:**
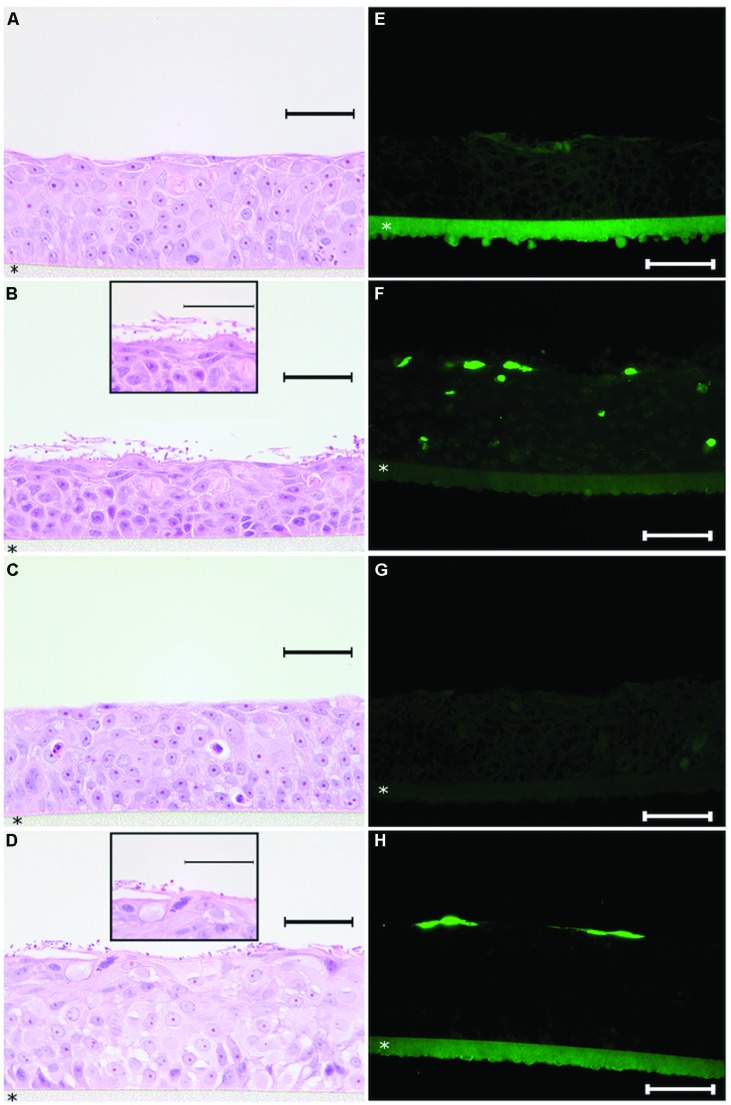
**Histopathology analysis of reconstructed human oral epithelium, exposed or not to histatin 5, both without *C. albicans*, exhibited a normal morphology **(A,C)** and no apoptotic cells (E,G)**. For the tissues exposed to *C. albicans*, hematoxylin and eosin (H&E) stained sections demonstrated efficient adherence of *C. albicans* on the outer layer of the reconstructed human oral epithelium (HOE; **B,D**). In the group which was not treated with histatin 5, a higher number of *C. albicans* in hyphae form **(B)** was observed. Interestingly, for the group treated with histatin 5 (50 μg ml^-1^), we can observe that *C. albicans* cells are in the non-invasive form **(D)**. For the TUNEL assay, HOE treated with histatin 5 showed presence of apoptotic cells restricted to the outer layer **(H)**. In contrast, the group without histatin 5 clearly displays the presence of apoptotic cells in different layers **(F)**. Scale bars = 50 μm. All pictures were taken with ×40 magnification with exception of the details presents in figure **(B,D)** that are ×100 magnification. HOE is formed on polycarbonate filter (*).

After identifying the minimal concentration range needed to coat a solid surface with histatin 5 in order to obtain a significant inhibition effect on *C. albicans* biofilm, our approach was to evaluate the effect of histatin 5 adsorbed to HOE cells using a similar concentration. **Figure 4** shows that untreated HOE (G1) and the group treated with 50 μg/mL of histatin 5 (G3), both without *C. albicans*, exhibited a normal HOE morphology (**Figures 4A,C**). Our results also demonstrated that the epithelium cells treated according to G1 and G3 are not in apoptotic stage (**Figures 4E,G**).

In order to assess the potential implications of histatin 5 as a protein that is able to protect the oral epithelium against microorganism adhesion, the tissues from G2 and G4 were infected with *C. albicans*. As expected, examination of H&E stained tissue sections demonstrated efficient adherence of *C. albicans* on the outer layer of the reconstructed HOE (**Figures 4B,D**). For G2, which was not treated with histatin 5, a higher number of *C. albicans* in hyphae form (**Figure 4B**) was observed. Interestingly, for the group treated with histatin 5 (G4), we can observe that *C. albicans* cells are in the non-invasive form. These images were consistent with those observed with TUNEL assay, where the group treated with histatin 5 showed that the presence of the apoptotic cells were restricted to the outer layer (**Figure 4H**). In contrast, the group without histatin 5 clearly displays the presence of apoptotic cells in different layers.

## Discussion

The processes that lead to the development of oral infections have been extensively studied. In the cases when there is balance between the virulence of a microorganism and the host ability to prevent microbial colonization, both host and microorganism can leave in a commensal state. On the other hand, when there is an imbalance, the oral cavity becomes an opportune environment for the development of infection diseases such as candidiasis, *C. albicans* being one of the most successful oral pathogens (Gow et al., 2012).

The fungicidal activity of histatin 5 has been shown to compromise residues 11–24 (Raj et al., 1990) where histatin 5 enters the *C. albicans* cell by involving specific receptors and/or driven by the transmembrane potential causing mitochondrial swelling, inhibiting the Krebs cycle, reducing the expression of an ATPase complex, and leading to a decrease in ATP production (Komatsu et al., 2011). The final result is the release of ATP and other essential energy storage molecules from the cell and cellular demise.

The data from killing assay (**Figure 1**) indicates that *C. albicans* are highly susceptible to histatin 5 solutions ranged from 800 to 25 μg/mL, leading to an approximated 2-log reduction in CFU mL^-1^. Our results are in accordance with Konopka et al. (2010), which treated different strains of *C. albicans* with histatin 5, and concluded that in a concentration of 4.8 μM, histatin 5 was able to reduce the yeast growth by 50%. In addition, it was observed that there is no statistical significant difference between the solutions ranged from 800 to 25 μg/mL. This observation might be due to the fact that histatin precipitates at > 64 μg/mL (Pettit et al., 2005). According to Jori et al. (2006), the efficiency of a treatment can be expressed when it induces a 4-log decrease in the survival of microorganisms; however, herein is an *in vitro* study, and in the oral cavity histatin 5 is secreted continuously.

The effect of histatin 5 when adsorpted to microtiter plate prior *C. albicans* biofilm formation (90 min, 24, 48, and 72 h) are summarized in **Figure 2**. The reduction of *C. albicans* was significantly higher for the group treated with histatin 5, irrespective of the evaluated time point (*p* = 0.00). Interestingly, 48 h histatin 5 coating resulted in a reduction of biofilm development compared to the 24 and 72 h, suggesting that histatin 5 is effective in reducing *C. albicans* growth during a later stage. According to Pusateri et al. (2009), PMMA disks treated with histatin 5 did not present an effect in reducing biofilm until 72 h. Similar results showed that the amount of *C. albicans* initially attached to PMMA surface was not significantly different between the control group and a group treated with histatin 5 until after 24 h (Yoshinari et al., 2006). However, herein, in the initial stage of *C. albicans* adhesion (90 min), a decrease in the number of cells was observed, showing a clear effect of histatin 5. In another study, histatin 5 adsorbed on PMMA or hydroxyapatite effectively inhibited *C. albicans* adhesion in initial stages and continued this inhibitory effect after 24 h (Vukosavljevic et al., 2012).

When histatin 5 was incubated for 24 h over a prior formed 48 h *C. albicans* biofilm, even with high concentrations of histatin 5, no effect against the *C. albicans* was observed (**Figure 3**). Development of *C. albicans* biofilms are associated with an increasing presence of extracellular polysaccharides (EPS), which is known to physically interact with antibiotics and contributes to resistance against these drugs Hawser and Douglas (1995). Moreover, *Candida* sp. EPS has a hydrophobic characteristic, which can diminish the penetration of histatin 5 into *C. albicans* biofilm (Chandra et al., 2001b).

Merely the presence of *C. albicans* cannot be related to the candidiasis establishment. The change of yeast to hyphae is a critical step for the host invasion by *C. albicans* and colonization of host tissue (Berman and Sudbery, 2002). Interestingly, our study demonstrated that pre exposition of histatin 5 to oral epithelial cells diminished the adhesion of *C. albicans* to the epithelium. In addition, a change to hyphae form was significantly inhibited when histatin 5 was adsorbed to HOE. This outcome suggests that histatin 5 interfere in the not totally characterized mechanism of *C. albicans* adhesion on the oral mucosa.

*Candida albicans* has the ability to invade and damage oral epithelial cells, which is critical for infection establishment. Indeed, oral epithelial cells after 18 h of candidal infection demonstrate significant death prevalence. The invasion for *C. albicans* stimulates oral epithelial signaling pathways and causes early apoptotic cell death, which is followed by secondary necrosis (Villar and Zhao, 2010). Our results verified that pre incubation of histatin 5 to oral epithelium drastically decreased the oral epithelium apoptosis caused by *C. albicans*, which was restricted to the outer layer of HOE. This event can most likely be explained by inhibition of hyphae formation when histatin 5 is adsorbed to HOE. In addition, only histatin 5 in the tested concentration did not damage the epithelial cell, which suggests a low cytotoxicity effect of this protein against host cell. Moreover, our cytotoxicity data showed that histatin 5 at the concentration of 50 μg/mL was able to cause an inhibition of 9% on cell viability of gingival fibroblast, and according to ISO standard 10993-5, a inhibition below 25% is considered not cytotoxic.

In the present study, different assays were used to quantify the activity of histatin 5 against *C. albicans* planktonic cells, biofilm and an *in vitro* formation of a histatin 5 oral mucosal pellicle. Histatin 5 in a physiological concentration was able to protect the HOE against *C. albicans* colonization and, at the same time, not interfere in the host cell homeostasis. This exciting outcome prepares a base for clinical research where the protection of the human oral mucosa against yeast infection could be evaluated by using a native protein.

## Conflict of Interest Statement

The authors declare that the research was conducted in the absence of any commercial or financial relationships that could be construed as a potential conflict of interest.
